# Non-reassuring fetal status: Case definition & guidelines for data collection, analysis, and presentation of immunization safety data

**DOI:** 10.1016/j.vaccine.2016.03.043

**Published:** 2016-12-01

**Authors:** Courtney Gravett, Linda O. Eckert, Michael G. Gravett, Donald J. Dudley, Elizabeth M. Stringer, Tresor Bodjick Muena Mujobu, Olga Lyabis, Sonali Kochhar, Geeta K. Swamy, The Brighton Collaboration Non-reassuring fetal status Working Group

**Affiliations:** a Global Alliance to Prevent Prematurity and Stillbirth, Seattle Children's Hospital, Seattle, USA; b University of Washington, USA; c University of Virginia, USA; d University of North Carolina, USA; e University of Kinshasa, The Democratic Republic of the Congo; f Sanofi Pasteur, Russia; g Global Healthcare Consulting, India; h Duke University, USA

**Keywords:** Non-reassuring fetal status, Fetal distress, Fetal intolerance to labor, Adverse event, Immunization, Guidelines, Case definition

## 1 Preamble

### 1.1 Need for developing case definitions and guidelines for data collection, analysis, and presentation for non-reassuring fetal status as an adverse event following immunization

Non-reassuring fetal status is a term used to describe suspected fetal hypoxia and is meant to replace the more ubiquitous term “fetal distress.” Fetal distress, defined as progressive fetal hypoxia and/or acidemia secondary to inadequate fetal oxygenation, is a term that is used to indicate changes in fetal heart patterns, reduced fetal movement, fetal growth restriction, and presence of meconium stained fluid [1]. Although fetal distress may be associated with neonatal encephalopathy, the generic term has poor predictive value for neonatal outcomes; most neonates will be vigorous and healthy at birth despite a diagnosis of fetal distress. Fetal distress can only be observed indirectly, usually via electronic fetal heart rate monitoring which is subject to high intra- and inter-observer variability in data interpretation [2], [3], [4]. For this reason, many experts recommend abandoning the term fetal distress, and adopting the term non-reassuring fetal status to describe clinical interpretation of fetal well-being [1], [5], [6]. Consistent with current opinion in the field, we recommend use of the term non-reassuring fetal status for use in monitoring fetal response following immunization.

Non-reassuring fetal status is not an adverse event per se, but rather an indicator of an underlying condition resulting in temporary or permanent oxygen deprivation to the fetus which may lead to fetal hypoxia and metabolic acidosis. Since fetal oxygenation is dependent upon maternal oxygenation and placental perfusion, perturbations of maternal oxygenation, uterine blood supply, placental transfer or fetal gas transport may lead to fetal hypoxia and non-reassuring fetal status [7]. Conditions commonly associated with non-reassuring fetal status include maternal cardiovascular disease, anemia, diabetes, hypertension, infection, placental abruption, abnormal presentation of the fetus, intrauterine growth restriction and umbilical cord compression, among other obstetric, maternal or fetal conditions.

The fetus experiences three stages of deterioration when oxygen levels are depleted: transient hypoxia without metabolic acidosis, tissue hypoxia with a risk of metabolic acidosis, and hypoxia with metabolic acidosis [7], [8]. Fetal response to oxygen deprivation is regulated by the autonomous nervous system, mediated by parasympathetic and sympathetic mechanisms. The fetus is equipped with compensatory mechanisms for transient hypoxia during labor, but prolonged, uninterrupted fetal hypoxia may lead progressively to acidosis with cell death, tissue damage, organ failure and potentially death. In response to hypoxia, fetal compensatory mechanisms include 1) a decrease in heart rate; 2) a reduction in oxygen consumption secondary to cessation of nonessential functions such as gross body movements; 3) a redistribution of cardiac output to preferentially perfuse organs, such as the heart, brain, and adrenal glands; and 4) a switch to anaerobic cellular metabolism [9]. Prolonged fetal hypoxia is associated with significant perinatal morbidity and mortality with particular concern for short- and long-term complications including encephalopathy, seizures, cerebral palsy, and neurodevelopmental delay [10], [11]. The fetal heart rate changes markedly in response to prolonged oxygen deprivation, making fetal heart rate monitoring a potentially valuable and commonly used tool for assessing fetal oxygenation status in real time. Non-reassuring fetal heart rate patterns are observed in approximately 15% of labors [12].

The two most common methods of monitoring fetal heart rate are cardiotocography (CTG) and intermittent auscultation. In high resource settings, continuous electronic fetal heart rate monitoring, via cardiotocography is the most prevalent method. Continuous CTG involves monitoring the fetal heart rate and uterine contractility simultaneously to detect fetal heart rate patterns associated with deficient fetal oxygen supply [8]. Normal CTG tracings are characterized by 1) stable baseline fetal heart rate (FHR) of 120–160 beats per minute (bpm), 2) FHR variability between 5 and 25 bpm above and below baseline FHR, and 3) periodic changes in the baseline FHR (accelerations above baseline or decelerations below baseline) [13]. While accelerations are associated with fetal well-being, decelerations, especially prolonged bradycardia, late decelerations, and severe variable decelerations are indicative of fetal stress and should prompt the clinician to evaluate and initiate intrauterine resuscitation with consideration for delivery of the fetus as indicated. Abnormal fetal heart rate patterns have high sensitivity, but low specificity and low predictive value to discriminate between neonates with or without metabolic acidosis [14]. While a normal fetal heart rate pattern is usually indicates reassuring fetal status, an abnormal fetal heart rate pattern does not necessarily equate with hypoxia or acidosis.

Although continuous CTG is the accepted standard of care in most high resource settings, use of continuous CTG in low resource setting is not feasible or recommended [15]. Continuous CTG requires costly equipment, expert maintenance, supply chain for consumables, and extensive training and high level of technical skill to interpret tracings. Additionally, continuous CTG can lead to higher rates of un-necessary interventions that may pose additional risk to mothers in settings where safe cesarean delivery is not readily available [16].

In settings where CTG is unavailable, intermittent auscultation is recommended for all laboring parturients [17]. Intermittent auscultation (IA) involves assessing the fetal heart rate at predetermined intervals with either a fetal stethoscope, or handheld Doppler. Abnormal heart rate findings by IA indicative of non-reassuring fetal status include prolonged fetal tachycardia or bradycardia, presence of repetitive or prolonged decelerations, and uterine tachysystole (more than 5 uterine contractions in a 10 min period). There is no evidence that IA performs worse than CTG in reducing morbidity and mortality associated with fetal acidosis. Studies comparing CTG to IA show no reduction in the risk of perinatal death or cerebral palsy [16], [18]. Intermittent auscultation, characterized by low cost and low technology equipment, is more feasible than CTG in low resource settings. However, it requires a high level of training and skill, frequent interaction between patient and health care provider, and does not provide as sophisticated a level of information that may be needed in high risk populations.

Several efforts to develop standards for defining non-reassuring fetal status have been made in response to confusion in recognizing and managing fetal heart rate patterns indicative of fetal compromise. The most widely accepted standards for classifying non-reassuring fetal heart rates come from the National Institute of Child Health and Human Development (NICHD) in the United States and The International Federation of Gynecology and Obstetrics (FIGO) (Table 1). In 1997, NICHD convened a workshop with the express purpose of developing “a standardized and rigorously, unambiguously described set of definitions to quantitate fetal heart monitoring [19]. The workshop produced standardized nomenclature for characterizing fetal heart rate patterns, which was widely adopted by western obstetric societies. In 2008, a follow-up workshop including the Society for Maternal Fetal Medicine and the American College of Obstetricians and Gynecologists was convened, resulting in the development of a three-tiered classification system of fetal heart rate patterns to guide management of fetal compromise [20], [21]. The Working Group uses these guidelines as a basis for the highest level of certainty for defining a case of non-reassuring fetal status. The NICHD guidelines are limited in defining fetal status in all settings as they are only intended for use with CTG. A second important set of guidelines, more applicable to all settings was first introduced by FIGO in 1986 and was updated in 2015 [17], [22], [23], [24], [25], [26]. The FIGO guidelines are the only guidelines with broad international consensus, and are simplified, with less emphasis on decelerations compared to the NICHD guidelines when evaluating CTG tracings. FIGO also provides recommendations for evaluating and categorizing fetal heart rate via intermittent auscultation, making these guidelines more useful for low resource settings. When CTG is not available, the Working Group recommends incorporating heart rate patterns from IA into the case definition for non-reassuring fetal status.

Little is known about the relationships among non-reassuring fetal status and maternal immunization, especially in LMIC where fetal assessment may not be routine. There are few publications reporting on fetal status following immunization; those that do are case reports or small series that have frequently not used standardized definitions [27], [28], [29]. Possible reasons that immunization surveillance has failed to report on cases of non-reassuring fetal status include the fact that a causal relationship is rarely if ever established, the low predictive value of non-reassuring fetal status to predict adverse neonatal outcomes, the difficulty of temporally associating vaccination with fetal status, as the two events are likely to be monitored at very different time-points in pregnancy, the failure to include fetal status as an outcome variable in immunization trials or surveillance. Estimates of the incidence of fetal status following maternal immunization have been hampered by limited data and lack of standard case definitions.

Moving forward, uniform and standardized definitions for non-reassuring fetal status and fetal well-being will be critical in immunization trials surveillance and monitoring to insure data comparability across trials. This is imperative to facilitate data interpretation and promote the scientific understanding of the event.

### 1.2 Methods for the development of the case definition and guidelines for data collection, analysis, and presentation for non-reassuring fetal status as an adverse events following immunization

Following the process described in the overview paper as well as on the Brighton Collaboration Website http://www.brightoncollaboration.org/internet/en/index/process.html, the Brighton Collaboration *Fetal Distress Working Group* was formed in 2015 and included members of clinical and academic, as well as public health background. Members have experience in high and low resources settings. The composition of the working and reference group as well as results of the web-based survey completed by the reference group with subsequent discussions in the working group can be viewed at: http://www.brightoncollaboration.org/internet/en/index/working_groups.html.

To guide the decision-making for the case definition and guidelines, literature was searched using Medline, Embase and the Cochrane Libraries, including the terms *vaccines, vaccination*, or *immunization* (*or terms beginning with vaccin-, immuni-, inocular*-) and *non-reassuring fetal status, fetal distress, intrapartum fetal asphyxia, non-reassuring fetal heart rate, fetal compromise, fetal hypoxia, fetal intolerance of labor*. To identify case definitions and measures of fetal distress in all settings, the above search terms were also searched with the terms *developing country* or *low resource setting*. The search was limited to publications written in English with human subjects. The search resulted in the identification of 105 references. All abstracts were screened for possible reports of non-reassuring fetal status, or fetal distress, following immunization. Eighteen articles with potentially relevant material were reviewed in more detail, in order to identify studies using case definitions or, in their absence, providing clinical descriptions of the case material. This resulted in a detailed summary of 2 articles, including information on the study type, the vaccine, the diagnostic criteria or case definition put forth, the time interval since immunization, and other symptoms. Multiple general medical and obstetric text books and obstetric society publications were also searched.

Most publications were single case reports. The terminology and case definitions were inconsistent among studies, with very few reporting case definitions at all. An inventory comprising 5 relevant case definitions of non-reassuring fetal status was made available to working group members.

### 1.3 Rationale for selected decisions about the case definition of non-reassuring fetal status as an adverse event following immunization

#### 1.3.1 The term non-reassuring fetal status

Several related terms are commonly used to describe fetal status including “fetal distress”, “birth asphyxia”, and “fetal intolerance to labor”. The Working Group was initially tasked with developing a case definition for “fetal distress”, but for reasons previously discussed, this term was abandoned and replaced with “non-reassuring fetal status”. The Working Group chose not to use the term “fetal intolerance of labor” because specifying such a narrow timeframe fails to capture non-reassuring fetal status in the antepartum period prior to the onset of labor.

In developing a case definition for non-reassuring fetal status, the Working Group included only cases for which fetal heart rate can be ascertained. The inability to measure fetal heart rate does not permit a diagnosis of non-reassuring fetal status at any acceptable level of diagnostic certainty. Within the definition context, however, the three diagnostic levels must not be misunderstood as reflecting different grades of clinical severity. They instead reflect diagnostic certainty (see below). All Levels are considered acceptable depending on the availability of diagnostic tools in each site.

#### 1.3.2 The term “birth asphyxia”

Birth asphyxia is often erroneously used interchangeably with fetal distress. Birth asphyxia is defined as the failure of the neonate to start regular respiration within one minute of birth, resulting from progressive hypoxia leading to acidosis in utero. Non-reassuring fetal status is distinct from birth asphyxia, as non-reassuring fetal status may be detected via fetal heart monitoring as a response to fetal hypoxia long before acidosis or asyphxia occur and will not necessarily result in birth asphyxia [5]. Therefore, although non-reassuring fetal status may be observed prior to birth asphyxia, the Working Group did not include this term in the developing the case definition.

#### 1.3.3 Formulating a case definition that reflects diagnostic certainty: weighing specificity versus sensitivity

As detection of non-reassuring fetal status is dependent on the type of technology used, and variable interpretation of fetal heart rate tracings, the evidence documenting fetal status may vary considerably. The case definition has been formulated such that the Level I definition is maximally specific for the condition, and relies on the highest level of evidence and technology available to detect the event. Two additional diagnostic levels have been included in the definition, offering a stepwise decrease in technological requirements, in an attempt to be inclusive of settings with less sophisticated means of detecting fetal well-being. In this way, our intent is that all possible cases of non-reassuring fetal status in all settings can be captured.

Importantly, the grading of definition levels is based entirely on diagnostic certainty, not clinical severity of an event. Thus, a clinically severe event may appropriately be classified as Level Two or Three rather than Level One if it could reasonably be due to non-reassuring fetal status. Detailed information about the severity of the event should additionally always be recorded, as specified by the data collection guidelines.

#### 1.3.4 The meaning of “sudden onset” and “rapid progression” in the context of non-reassuring fetal status

The term “sudden onset” refers to an event that occurred unexpectedly and without warning leading to a marked change in a woman's previously stable condition.

The term “rapid progression” is a conventional clinical term. An exact time-frame should not be offered since this could refer to a wide range of signs and symptoms without a scientific evidence base. Using an arbitrarily restrictive set point might bias future data collection unnecessarily.

#### 1.3.5 Rationale for individual criteria or decision made related to the case definition

##### 1.3.5.1 Radiology findings

Doppler sonography utilizes ultrasound to measure the change in frequency of energy wave transmission when relative motion occurs between the source and the observer. Doppler sonography is used for non-invasive assessment of circulation in many clinical conditions. In obstetrics, fetal umbilical artery (UA) doppler velocimetry provides a noninvasive measure of the fetoplacental hemodynamic state. Abnormal UA Doppler indices indirectly reflect impedance of downstream circulation and have been associated with fetal hypoxia, fetal acidosis, and adverse perinatal outcomes [30], [31], [32], [33]. Randomized trials integrating UA Doppler velocimetry into antepartum fetal surveillance of high risk pregnancies have demonstrated efficacy in the setting of fetal growth restriction or preeclampsia [34]. In addition to UA Doppler, middle cerebral artery (MCA) Doppler is used in the setting of suspected fetal anemia. Obstetric Doppler imaging requires a high level of training and technical ultrasound equipment, is not usually performed during labor when CTG is the preferred and superior modality to assess fetal well-being, and is not suitable for low resource settings. For these reasons, the Working Group decided that Doppler sonography does not merit inclusion in the case definition of non-reassuring fetal status.

##### 1.3.5.2 Laboratory findings

###### 1.3.5.2.1 Fetal blood sampling

Discontinuous fetal blood sampling has been used to monitor fetal acid–base metabolism in the intrapartum period, when fetal heart rate tracings are suggestive of hypoxic insult. Fetal blood sampling allows analysis of pH, lactate concentrations, partial pressure oxygen (*p*O_2_) and carbon dioxide (*p*CO_2_) from which base excess is calculated (BE). Fetal blood pH less than or equal to 7.20, *p*O_2_ > 65 mmHg, and BE > −9.8, and lactate >4.8 mmol/L indicate metabolic acidosis requiring intervention to restore adequate oxygen supply to the fetus [26], [35], [36]. Fetal blood sampling is problematic in diagnosing non-reassuring fetal status for several reasons. Fetal blood sampling is an invasive and uncomfortable procedure, requiring ruptured membranes and incision into the fetal scalp to sample blood, thus exposing the fetus to risk of infection [37]. The procedure requires a high level of training and technical laboratory equipment including real time blood gas analysis [38]. Fetal blood sampling is seldom performed in high resource settings and is not suitable for low resource settings. For these reasons, the Working Group concluded that fetal blood sampling does not merit inclusion in the case definition of non-reassuring fetal status.

###### 1.3.5.2.2 Umbilical cord blood sampling

Immediately following birth, metabolic acidosis in the fetus can be detected by analyzing arterial and venous blood from the umbilical cord. Cord blood analysis for pH, *p*CO_2_, and the derivative bicarbonate (HCO_3_) and base deficit (BD) values is highly recommended in all cases of suspected fetal hypoxia/acidosis. Metabolic acidosis is defined as the measurement of the umbilical artery blood pH < 7.00 and BD > 12 mmol/L [39]. The Working Group included cord blood analysis as part of the case definition for non-reassuring fetal status as the only method to objectively ascertain the occurrence of fetal hypoxia/acidosis immediately prior to birth. Recognizing that not all settings will be equipped to perform cord blood analysis, the working group did not include these criteria across all levels of diagnostic certainty.

###### 1.3.5.2.3 Pathology findings: autopsy

The term non-reassuring fetal status does not have high positive predictive value for neonatal morbidity and mortality. Furthermore, non-reassuring fetal status does not produce pathognomonic post-mortem features. Therefore, post-mortem findings are not included in the case definition of non-reassuring fetal status.

###### 1.3.5.2.4 Physical findings

The APGAR score is a standardized assessment of the neonate's physiological condition immediately following birth, as well the neonatal response to resuscitation, if required [40]. The APGAR score evaluates color, heart rate, reflexes, muscle tone, and respiration. Low APGAR scores may be observed as a result of intrapartum fetal compromise. Fetal hypoxic insult may precede low APGAR scores when the hypoxic injury is sufficient to affect the pulmonary, neurologic or cardiovascular system of the fetus, but APGAR scores alone are not sufficiently sensitive or specific for diagnosing fetal hypoxia or acidosis and are only weakly associated with non-reassuring fetal heart rate patterns. There is a low correlation between low 1 and 5 min APGAR scores and metabolic acidosis [41], [42], but many other conditions, including neonatal sepsis, trauma, maternal drug use, fetal anomalies and gestational age that may contribute to low APGAR scores as well, making causal inference problematic [8]. Furthermore, APGAR scoring in not well standardized, and is inconsistently used in the global setting. The Working Group did not include APGAR scores as part of the case definition for non-reassuring status, but we do recommend that 1, 5, and 10 min APGAR scores be collected for vaccine monitoring and surveillance purposes.

#### 1.3.6 Influence of treatment on fulfillment of case definition

The Working Group decided against using “treatment” or “treatment response” toward fulfillment of the non-reassuring fetal status case definition. A treatment response or failure is not in itself diagnostic, and may depend on variables such as clinical status, time to treatment, and other clinical parameters. Treatment strategies for non-reassuring fetal status are variable, ranging from intrauterine resuscitation to performing cesarean section for immediate delivery of a compromised fetus. Treatment response will vary depending on the severity of fetal heart rate patterns, the duration of altered heart rate, the clinician's assessment of these factors, and the resources available for intervention.

#### 1.3.7 Timing post immunization

Specific time frames for onset of symptoms following immunization are not included for the following reasons:

Time from immunization was not included for defining non-reassuring fetal status because fetal status may change at unknown periods in the ante- or intra-partum periods. Although we recognize fetal status is most likely to be observed in the intrapartum period when fetal heart rate monitoring is deployed, we did not want to narrow the time frame to the exclusion of fetal events in the antepartum period. The Working Group does recommend that the time elapsed from vaccine administration to observation of non-reassuring fetal status be recorded as a critical variable for data collection.

We postulate that a definition designed to be a suitable tool for testing causal relationships requires ascertainment of the outcome (e.g. non-reassuring fetal status) independent from the exposure (e.g. immunizations). Therefore, to avoid selection bias, a restrictive time interval from immunization to onset of non-reassuring fetal status should not be an integral part of such a definition. Instead, where feasible, details of this interval should be assessed and reported as described in the data collection guidelines.

Further, non-reassuring fetal status often occurs outside the controlled setting of a clinical trial or hospital. In some settings determining a clear timeline of the event may be impossible, particularly in less developed or rural settings. In order to avoid selecting against such cases, the Brighton Collaboration case definition avoids setting arbitrary time frames.

### 1.4 Guidelines for data collection, analysis and presentation

As mentioned in the overview, the case definition is accompanied by guidelines which are structured according to the steps of conducting a clinical trial, i.e. data collection, analysis and presentation. Neither case definition nor guidelines are intended to guide or establish criteria for management of ill infants, children, or adults. Both were developed to improve data comparability.

### 1.5 Periodic review

Similar to all Brighton Collaboration case definitions and guidelines, review of the definition with its guidelines is planned on a regular basis (i.e. every three to five years) or more often if needed.

## 2 Case definition of non-reassuring fetal status

Level 1 of diagnostic certainty

• Category III fetal heart rate tracings detected via continuous cardiotocography as defined by NICHD [20]
∘ Absent baseline fetal heart rate variability AND any of the following:
- recurrent late decelerations
- recurrent variable deceleration
- bradycardia (<110 bpm)
  OR
∘ Sinusoidal pattern
  AND
• Umbilical cord blood analysis consistent with metabolic acidosis (pH < 7.0 and Base deficit >12 mmol/L)

Level 2 of diagnostic certainty

• Category III fetal heart rate tracings detected via continuous cardiotocography as defined by NICHD [20]
∘ Absent baseline fetal heart rate variability AND any of the following:
- recurrent late decelerations
- recurrent variable deceleration
- bradycardia (<110 bpm)
  OR
∘ Sinusoidal pattern

Level 3 of diagnostic certainty

• Fetal heart pattern detected via intermittent auscultation suggestive of fetal hypoxia [17]
∘ Baseline FHR <110 bpm or >160 bpm
∘ Presence of repetitive or prolonged (>3 min) decelerations
∘ More than 5 contractions in a 10 min period

**Major and minor criteria used in the case definition of non-reassuring fetal status**

**Major criteria**

Cardiovascular

CTG:

Category III heart

Rate tracing

• Absent baseline fetal heart rate variability AND any of the following:
- recurrent late decelerations
- recurrent variable deceleration
- bradycardia < 110 beats/min
  OR
• Sinusoidal pattern

IA:

Abnormal

Findings

• FHR <110 bpm OR >160 bpm
• Presence of repetitive or prolonged decelerations
• More than 5 contractions in a 10 min period

**Minor criteria**

Laboratory

• Cord blood pH ≤7.0
• Cord blood base deficit ≥12 mmol

## 3 Guidelines for data collection, analysis and presentation of non-reassuring fetal status

The consensus of the Brighton Collaboration *Non-Reassuring Fetal Distress Working Group* for non-reassuring fetal status was to recommend the following guidelines to enable meaningful and standardized collection, analysis, and presentation of information about non-reassuring fetal status. However, implementation of all guidelines might not be possible in all settings. The availability of information may vary depending upon resources, geographical region, and whether the source of information is a prospective clinical trial, a post-marketing surveillance or epidemiological study, or an individual report of non-reassuring fetal status. Also, as explained in more detail in the overview paper in this volume, these guidelines have been developed by this working group for guidance only, and are not to be considered a mandatory requirement for data collection, analysis, or presentation.

### 3.1 Data collection

These guidelines represent a desirable standard for the collection of data on availability following immunization to allow for comparability of data, and are recommended as an addition to data collected for the specific study question and setting. The guidelines are not intended to guide the primary reporting of non-reassuring fetal status to a surveillance system or study monitor. Investigators developing a data collection tool based on these data collection guidelines also need to refer to the criteria in the case definition, which are not repeated in these guidelines. The Brighton Collaboration has developed guidelines for data collection https://brightoncollaboration.org/public/resources/standards/guidelines.html; and data collection forms https://brightoncollaboration.org/public/resources/data-collection-forms.html.

Guidelines numbers below have been developed to address data elements for the collection of adverse event information as specified in general drug safety guidelines by the International Conference on Harmonization of Technical Requirements for Registration of Pharmaceuticals for Human Use (43), and the form for reporting of drug adverse events by the Council for International Organizations of Medical Sciences (44). These data elements include an identifiable reporter and patient, one or more prior immunizations, and a detailed description of the adverse event, in this case, of non-reassuring fetal status following immunization. The additional guidelines have been developed as guidance for the collection of additional information to allow for a more comprehensive understanding of non-reassuring fetal status following immunization.

#### 3.1.1 Source of information/reporter

For all cases and/or all study participants, as appropriate, the following information should be recorded:

1) Date of report.
2) Name and contact information of person reporting^2^ and/or diagnosing the non-reassuring fetal status as specified by country-specific data protection law.
3) Name and contact information of the investigator responsible for the patient, as applicable.
4) Relation to the patient (e.g., immunizer [clinician, nurse], family member [indicate relationship], other).

#### 3.1.2 Vaccinee/Control

##### 3.1.2.1 Demographics

For all cases and/or all study participants, as appropriate, the following information should be recorded:

5) Case/study participant identifiers (e.g. first name initial followed by last name initial) or code (or in accordance with country-specific data protection laws).
6) Date of birth, age, and sex.

##### 3.1.2.2 Clinical and immunization history

For all cases and/or all study participants, as appropriate, the following information should be recorded:

7) Past medical history, including hospitalizations, underlying diseases/disorders, pre-immunization signs and symptoms including identification of indicators for, or the absence of, a history of allergy to vaccines, vaccine components or medications; food allergy; allergic rhinitis; eczema; asthma.
8) Any medication history (other than treatment for the event described) prior to, during, and after immunization including prescription and non-prescription medication as well as medication or treatment with long half-life or long term effect. (e.g. immunoglobulins, blood transfusion and immunosuppressants).
9) Immunization history (i.e. previous immunizations and any adverse event following immunization (AEFI)), in particular occurrence of non-reassuring fetal status after a previous immunization.

#### 3.1.3 Details of the immunization

For all cases and/or all study participants, as appropriate, the following information should be recorded:

10) Date and time of immunization(s).
11) Description of vaccine(s) (name of vaccine, manufacturer, lot number, dose (e.g. 0.25 mL, 0.5 mL, etc.), composition of any diluent administered separately or added to the vaccine, and number of dose if part of a series of immunizations against the same disease).
12) The anatomical sites (including left or right side) of all immunizations (e.g. vaccine A in proximal left lateral thigh, vaccine B in left deltoid).
13) Route and method of administration (e.g. intramuscular, intradermal, subcutaneous, and needle-free (including type and size), other injection devices).
14) Needle length and gauge.

#### 3.1.4 The adverse event

15) For all cases at any level of diagnostic certainty and for reported events with insufficient evidence, the criteria fulfilled to meet the case definition should be recorded.

Specifically document:

16) Clinical description of signs and symptoms of non-reassuring fetal status, and if there was medical confirmation of the event (i.e. patient seen by physician).
17) Date/time of onset,^3^ first observation^4^ and diagnosis,^5^ end of episode^6^ and final outcome.^7^
18) Concurrent signs, symptoms, and diseases (e.g. maternal conditions, known fetal conditions, abnormalities of labor, abnormalities of delivery).
19) Time interval since immunization
20) Measurement/testing

• Values and units of routinely measured parameters (paper tracings of EFM) (e.g. temperature, blood pressure) – in particular those indicating the severity of the event;
• Method of measurement (e.g. cardiotocograph, doppler, fetoscope, etc. Include units of fetal heart trace (1 cm/min, etc.));
• Results of laboratory examinations, surgical and/or pathological findings and diagnoses if present (cord blood gases, pH)
• APGARS at 1, 5, 10 min for neonate
• Occurrence of neonatal seizures

Treatment given for non-reassuring fetal status, especially specify what and dosing.Outcome^6^ at last observation.Objective clinical evidence supporting classification of the event as “serious” 8 (e.g. 10 min APGAR of 3 or less; presence of neonatal seizures, newborn resuscitation required)Exposures other than the immunization 24 h before and after immunization (e.g. food, environmental, placental abruption, abdominal trauma) considered potentially relevant to the reported event.Neonatal disposition

• Gestational age
• Birth weight
• Birth outcome (e.g., live birth, stillbirth)
• Delivery method (e.g. spontaneous vaginal, assisted vaginal, cesarean section)
• 1, 5 and 10 min APGAR scores
• Presence of meconium

#### 3.1.5 Miscellaneous/General

26) The duration of surveillance for non-reassuring fetal status should be predefined based on

• Biologic characteristics of the vaccine e.g. live attenuated versus inactivated component vaccines;
• Biologic characteristics of the vaccine-targeted disease;
• Biologic characteristics of non-reassuring fetal heart rate including patterns identified in previous trials (e.g. early-phase trials); and
• Biologic characteristics of the vaccinee (e.g. nutrition, underlying disease like immunodepressing illness).

27) The duration of follow-up reported during the surveillance period should be predefined with continued follow-up to resolution of the event. For non-reassuring fetal status, the follow-up period should continue through the ante- and intra-partum periods, as changes in fetal well-being can occur at any point in pregnancy.
28) Methods of data collection should be consistent within and between study groups, if applicable.
29) Follow-up of cases should attempt to verify and complete the information collected as outlined in data collection guidelines 1–24.
30) Investigators of patients with non-reassuring fetal status should provide guidance to reporters to optimize the quality and completeness of information provided.
31) Reports of non-reassuring fetal status should be collected throughout the study period regardless of the time elapsed between immunization and the adverse event. If this is not feasible due to the study design, the study periods during which safety data are being collected should be clearly defined.

### 3.2 Data analysis

The following guidelines represent a desirable standard for analysis of data on non-reassuring fetal status to allow for comparability of data, and are recommended as an addition to data analyzed for the specific study question and setting.

32) Reported events should be classified in one of the following five categories including the three levels of diagnostic certainty. Events that meet the case definition should be classified according to the levels of diagnostic certainty as specified in the case definition. Events that do not meet the case definition should be classified in the additional categories for analysis.

**Event classification in 5 categories**^9^

**Event meets case definition**

1) Level 1: Criteria as specified in the non-reassuring fetal status case definition
2) Level 2: Criteria as specified in the non-reassuring fetal status case definition
3) Level 3: Criteria as specified in the non-reassuring fetal status case definition

**Event does not meet case definition**

***Additional categories for analysis***

4) Reported non-reassuring fetal status with insufficient evidence to meet the case definition^10^
5) Not a case of non-reassuring fetal status

33) The interval between immunization and reported non-reassuring fetal status could be defined as the date/time of immunization to the date/time of onset^2^ of the first symptoms and/or signs consistent with the definition. If few cases are reported, the concrete time course could be analyzed for each; for a large number of cases, data can be analyzed in the following increments:

**Subjects with non-reassuring fetal status by interval to presentation**

**Table:** 

Interval^*^	**Number**
<1 h after immunization	
1 h–<7 days after immunization	
7 days–<30 days after immunization	
>30 days–delivery after immunization	
**Total**

34) The duration of a possible non-reassuring fetal status could be analyzed as the interval between the date/time of onset^1^ of the first symptoms and/or signs consistent with the definition and the end of episode^5^ and/or final outcome.^6^ Whatever start and ending are used, they should be used consistently within and across study groups.
35) If more than one measurement of a particular criterion is taken and recorded, the value corresponding to the greatest magnitude of the adverse experience could be used as the basis for analysis. Analysis may also include other characteristics like qualitative patterns of criteria defining the event.
36) The distribution of data (as numerator and denominator data) could be analyzed in predefined increments (e.g. measured values, times), where applicable. Increments specified above should be used. When only a small number of cases is presented, the respective values or time course can be presented individually.
37) Data on non-reassuring fetal status obtained from subjects receiving a vaccine should be compared with those obtained from an appropriately selected and documented control group(s) to assess background rates of hypersensitivity in non-exposed populations, and should be analyzed by study arm and dose where possible, e.g. in prospective clinical trials.

### 3.3 Data presentation

These guidelines represent a desirable standard for the presentation and publication of data on non-reassuring fetal status following immunization to allow for comparability of data, and are recommended as an addition to data presented for the specific study question and setting. Additionally, we recommended to referring to existing general guidelines for the presentation and publication of randomized controlled trials, systematic reviews, and meta-analyses of observational studies in epidemiology (e.g. statements of Consolidated Standards of Reporting Trials (CONSORT), of Improving the quality of reports of meta-analyses of randomized controlled trials (QUORUM), and of Meta-analysis Of Observational Studies in Epidemiology (MOOSE), respectively) (45–47).

38) All reported events of non-reassuring fetal status should be presented according to the categories listed in guideline 31 (verify numbers).
39) Data on possible non-reassuring fetal status events should be presented in accordance with data collection guidelines 1–24 (verify numbers) and data analysis guidelines 31–36 (verify numbers).
40) Terms to describe non-reassuring fetal status such as “low-grade”, “mild”, “moderate”, “high”, “severe” or “significant” are highly subjective, prone to wide interpretation, and should be avoided, unless clearly defined.
41) Data should be presented with numerator and denominator (n/N) (and not only in percentages), if available.

Although immunization safety surveillance systems denominator data are usually not readily available, attempts should be made to identify approximate denominators. The source of the denominator data should be reported and calculations of estimates be described (e.g. manufacturer data like total doses distributed, reporting through Ministry of Health, coverage/population based data, etc.).

42) The incidence of cases in the study population should be presented and clearly identified as such in the text.
43) If the distribution of data is skewed, median and range are usually the more appropriate statistical descriptors than a mean. However, the mean and standard deviation should also be provided.
44) Any publication of data on non-reassuring fetal status should include a detailed description of the methods used for data collection and analysis as possible. It is essential to specify:

• The study design;
• The method, frequency and duration of monitoring for non-reassuring fetal status;
• The trial profile, indicating participant flow during a study including drop-outs and withdrawals to indicate the size and nature of the respective groups under investigation;
• The type of surveillance (e.g. passive or active surveillance);
• The characteristics of the surveillance system (e.g. population served, mode of report solicitation);
• The search strategy in surveillance databases;
• Comparison group(s), if used for analysis;
• The instrument of data collection (e.g. standardized questionnaire, diary card, report form);
• Whether the day of immunization was considered “day one” or “day zero” in the analysis;
• Whether the date of onset^2^ and/or the date of first observation^3^ and/or the date of diagnosis^4^ was used for analysis; and
• Use of this case definition for non-reassuring fetal status, in the abstract or methods section of a publication.^11^

## Disclaimer

The findings, opinions and assertions contained in this consensus document are those of the individual scientific professional members of the working group. They do not necessarily represent the official positions of each participant's organization (e.g., government, university, or corporation). Specifically, the findings and conclusions in this paper are those of the authors and do not necessarily represent the views of their respective institutions.

## Figures and Tables

**Table 1:** Comparison of NICHD and FIGO guidelines for interpretation of fetal heart rate via continuous cardiotocography. FHR = fetal heart rate, bpm = beats per minute.

NICHD three-tier fetal heart rate interpretation system (2008)		FIGO consensus guidelines on intrapartum fetal monitoring CTG tracing classifications (2015)	
FHR designation	Description	FHR designation	Description
Category I tracing	*Baseline heart rate*: 110–160 bpm *Variability*: Moderate *Decelerations*: No late decelerations Early decelerations may be present or absent Accelerations may be present or absent	Normal	*Baseline heart rate*: 110–160 bpm *Variability*: 5–25 bpm *Decelerations*: No repetitive decelerations
Category II tracing	FHR tracing does not meet criteria for category I or category III	Suspicious	Lacking at least one characteristic of normality, but with no pathologic features
Category III tracing	1) *Variability:* Absent FHR baseline variability AND any of the following: Recurrent late decelerations Recurrent variable decelerations Bradycardia (FHR < 110 bpm) OR 2) Sinusoidal pattern	Pathological	*Baseline heart rate*: <100 bpm *Variability*: Reduced variability for >15 min Increased variability for >30 min OR Sinusoidal pattern for >30 min *Decelerations*: Repetitive late or prolonged decelerations during >30 min or 20 min if reduced variability OR One prolonged deceleration with >5 min

## Footnotes

2 If the reporting center is different from the vaccinating center, appropriate and timely communication of the adverse event should occur.

3 The date and/or time of onset is defined as the time post immunization, when the first sign or symptom indicative for non-reassuring fetal status occurred. This may only be possible to determine in retrospect.

4 The date and/or time of first observation of the first sign or symptom indicative for non-reassuring fetal status can be used if date/time of onset is not known.

5 The date of diagnosis of an episode is the day post immunization when the event met the case definition at any level.

6 The end of an episode is defined as the time the event no longer meets the case definition at the lowest level of the definition.

7 E.g. recovery to pre-immunization health status, spontaneous resolution, therapeutic intervention, persistence of the event, sequelae, death.

8 An AEFI is defined as serious by international standards if it meets one or more of the following criteria: 1) it results in death, 2) is life-threatening, 3) it requires inpatient hospitalization or results in prolongation of existing hospitalization, 4) results in persistent or significant disability/incapacity, 5) is a congenital anomaly/birth defect, 6) is a medically important event or reaction.

9 To determine the appropriate category, the user should first establish, whether a reported event meets the criteria for the lowest applicable level of diagnostic certainty, e.g. Level three. If the lowest applicable level of diagnostic certainty of the definition is met, and there is evidence that the criteria of the next higher level of diagnostic certainty are met, the event should be classified in the next category. This approach should be continued until the highest level of diagnostic certainty for a given event could be determined. Major criteria can be used to satisfy the requirement of minor criteria. If the lowest level of the case definition is not met, it should be ruled out that any of the higher levels of diagnostic certainty are met and the event should be classified in additional categories four or five.

10 If the evidence available for an event is insufficient because information is missing, such an event should be categorized as “Reported non-reassuring fetal status with insufficient evidence to meet the case definition”. An event does not meet the case definition if investigation reveals a negative finding of a necessary criterion (necessary condition) for diagnosis. Such an event should be rejected and classified as “Not a case of non-reassuring fetal status”.

11 Use of this document should preferably be referenced by referring to the respective link on the Brighton Collaboration website (http://www.brightoncollaboration.org).

## References

[1] Parer J.T., Livingston E.G. (1990). What is fetal distress?. Am J Obstet Gynecol.

[2] Bernardes J., Costa-Pereira A., Ayres-de-Campos D., van Geijn H.P., Pereira-Leite L. (1997). Evaluation of interobserver agreement of cardiotocograms. Int J Gynaecol Obstet Off Organ Int Federat Gynaecol Obstet.

[3] Blackwell S.C., Grobman W.A., Antoniewicz L., Hutchinson M., Gyamfi Bannerman C. (2011). Interobserver and intraobserver reliability of the NICHD 3-Tier Fetal Heart Rate Interpretation System. Am J Obstet Gynecol.

[4] Rhose S., Heinis A.M., Vandenbussche F., van Drongelen J., van Dillen J. (2014). Inter- and intra-observer agreement of non-reassuring cardiotocography analysis and subsequent clinical management. Acta Obstet Gynecol Scand.

[5] Penning S., Garite T.J. (1999). Management of fetal distress. Obstet Gynecol Clin N Am.

[6] Practice ACoO. (2005). Inappropriate use of the terms fetal distress and birth asphyxia, Committee Opinion 326. Obstet Gynecol.

[7] Martin C.B. (2008). Normal fetal physiology and behavior, and adaptive responses with hypoxemia. Sem Perinatol.

[8] Murray M. (2007). Antepartal and intrapartal fetal monitoring.

[9] Fahey J., King T.L. (2005). Intrauterine asphyxia: clinical implications for providers of intrapartum care. J Midw Womens Health.

[10] (2014). Executive summary: neonatal encephalopathy and neurologic outcome, second edition, report of the American College of Obstetricians and Gynecologists’ Task Force on Neonatal Encephalopathy. Obstet Gynecol.

[11] Hankins G.D., Speer M. (2003). Defining the pathogenesis and pathophysiology of neonatal encephalopathy and cerebral palsy. Obstet Gynecol.

[12] East C.E., Leader L.R., Sheehan P., Henshall N.E., Colditz P.B., Lau R. (2015). Intrapartum fetal scalp lactate sampling for fetal assessment in the presence of a non-reassuring fetal heart rate trace. Cochrane Database Syst Rev.

[13] Ugwumadu A. (2013). Understanding cardiotocographic patterns associated with intrapartum fetal hypoxia and neurologic injury. Best Pract Res Clin Obstet Gynaecol.

[14] Low J.A., Victory R., Derrick E.J. (1999). Predictive value of electronic fetal monitoring for intrapartum fetal asphyxia with metabolic acidosis. Obstet Gynecol.

[15] Hofmeyr G.J., Haws R.A., Bergstrom S., Lee A.C., Okong P., Darmstadt G.L. (2009). Obstetric care in low-resource settings: what, who, and how to overcome challenges to scale up?. Int J Gynaecol Obstet Off Organ Int Federat Gynaecol Obstet.

[16] Alfirevic Z., Devane D., Gyte G.M. (2006). Continuous cardiotocography (CTG) as a form of electronic fetal monitoring (EFM) for fetal assessment during labour. Cochrane Database Syst Rev.

[17] Lewis D., Downe S. (2015). panel. Fifmc. FIGO consensus guidelines on intrapartum fetal monitoring: intermittent auscultation. Int J Gynecol Obstet.

[18] Alfirevic Z., Devane D., Gyte G.M. (2013). Continuous cardiotocography (CTG) as a form of electronic fetal monitoring (EFM) for fetal assessment during labour. Cochrane Database Syst Rev.

[19] (1997). Electronic fetal heart rate monitoring: research guidelines for interpretation. National Institute of Child Health and Human Development Research Planning Workshop. Am J Obstet Gynecol.

[20] Macones G.A., Hankins G.D., Spong C.Y., Hauth J., Moore T. (2008). The 2008 National Institute of Child Health and Human Development workshop report on electronic fetal monitoring: update on definitions, interpretation, and research guidelines. Obstet Gynecol.

[21] American College of Gynecologists (2010). Practice bulletin no. 116: management of intrapartum fetal heart rate tracings. Obstet Gynecol.

[22] Ayres-de-Campos D., Sabaratnam A. (2015). panel. Fifmec. FIGO consensus guidelines on intrapartum fetal monitoring: introduction. Int J Gynecol Obstet.

[23] Ayres-de-Campos D., Sabaratnam A. (2015). panel. Fifmec. FIGO consensus guidelines on intrapartum fetal monitoring: physiology of fetal oxygenation and the main goals of intrapartum fetal monitoring. Int J Gynecol Obstet.

[24] Ayres-de-Campo D., Spong C.Y., Chandraharan E. (2015). panel. Fifmec. FIGO consensus guidelines on intrapartum fetal monitoring: cardiotocography. Int J Gynecol Obstet.

[25] Medicine FsoSiP. (1987). Guidelines for the use of fetal monitoring. Int J Gynecol Obstet.

[26] Visser G., Ayres-de-Campos D. (2015). panel. Fifmec. FIGO consensus guidelines on intrapartum fetal monitoring: adjunctive technologies. Int J Gynecol Obstet.

[27] Adedinsewo D.A., Noory L., Bednarczyk R.A., Steinhoff M.C., Davis R., Ogbuanu C. (2013). Impact of maternal characteristics on the effect of maternal influenza vaccination on fetal outcomes. Vaccine.

[28] Donegan K., King B., Bryan P. (2014). Safety of pertussis vaccination in pregnant women in UK: observational study. BMJ.

[29] Munoz F.M., Bond N.H., Maccato M., Pinell P., Hammill H.A., Swamy G.K. (2014). Safety and immunogenicity of tetanus diphtheria and acellular pertussis (Tdap) immunization during pregnancy in mothers and infants: a randomized clinical trial. JAMA.

[30] Karsdorp V.H., van Vugt J.M., van Geijn H.P., Kostense P.J., Arduini D., Montenegro N. (1994). Clinical significance of absent or reversed end diastolic velocity waveforms in umbilical artery. Lancet.

[31] Maulik D., Maulik D. (2005). Absent end diastolic velocity in the umbilical artery and its clinical significance. Doppler ultrasound in obstetrics and gynecology.

[32] Valcamonico A., Danti L., Frusca T., Soregaroli M., Zucca S., Abrami F. (1994). Absent end-diastolic velocity in umbilical artery: risk of neonatal morbidity and brain damage. Am J Obstet Gynecol.

[33] Yoon B.H., Romero R., Roh C.R., Kim S.H., Ager J.W., Syn H.C. (1993). Relationship between the fetal biophysical profile score, umbilical artery Doppler velocimetry, and fetal blood acid–base status determined by cordocentesis. Am J Obstet Gynecol.

[34] Alfirevic Z., Stampalija T., Gyte G.M. (2013). Fetal and umbilical Doppler ultrasound in high-risk pregnancies. Cochrane Database Syst Rev.

[35] Hopp H., Nonnenmacher A. (2008). Evidence-based fetal assessment. Gynakol Geburtsmed Gynakol Endokrinol.

[36] Saling E. (1964). Blood gas relations and the acid–base equilibrium of the fetus in an uncomplicated course of delivery. Zeitschrift Geburtshilfe Gynakologie.

[37] Maiques V., Garcia-Tejedor A., Perales A., Navarro C. (1999). Intrapartum fetal invasive procedures and perinatal transmission of HIV. Eur J Obstet Gynecol Reprod Biol.

[38] Jibodu O.A., Arulkumaran S. (2000). Intrapartum fetal surveillance. Curr Opin Obstet Gynecol.

[39] Practice ACoO. (2006). ACOG Committee Opinion No. 348, November 2006: umbilical cord blood gas and acid–base analysis. Obstet Gynecol.

[40] Apgar V. (1953). A proposal for a new method of evaluation of the newborn infant. Curr Res Anes Anal.

[41] Leuthner S.R., Das U.G. (2004). Low Apgar scores and the definition of birth asphyxia. Pediatr Clin N Am.

[42] Manganaro R., Mami C., Gemelli M. (1994). The validity of the Apgar scores in the assessment of asphyxia at birth. Eur J Obstet Gynecol Reprod Biol.

